# Zinc Transporters and MTF1-Notch1-P21 Signalling Axis in TPEN-Induced Cell Death in Human Skeletal Muscle (Rhabdomyosarcoma) Cells

**DOI:** 10.7759/cureus.90420

**Published:** 2025-08-18

**Authors:** Kiran K Alluri, Mahesh Malleswarapu, Naga Babu Pyadala

**Affiliations:** 1 Biochemistry, MNR Medical College and Hospital, Hyderabad, IND; 2 Life Sciences, University of Hyderabad, Hyderabad, IND

**Keywords:** mtf1, notch1, p-21 protein, rhabdomyosarcoma (rd) cells, zinc transporter 1

## Abstract

Background

Physiological zinc levels play a crucial role in regulating cell viability and proliferation. Muscle tissue, a major zinc reservoir, is generally resistant to fluctuations in zinc concentration; however, zinc status can influence muscle cell duplication and survival. Zinc homeostasis is regulated by zinc transporters and signaling pathways such as Notch1 and PI3K/AKT, which control the expression of p21, a key regulator of cell cycle progression and apoptosis. This study aimed to investigate the impact of zinc status on human rhabdomyosarcoma (RD) cells and elucidate the involvement of the metal-regulatory transcription factor 1 (MTF1)-Notch1-PI3K/AKT-p21 axis in zinc depletion-induced cell death.

Methodology

Zinc depletion was induced in RD cells by treatment with 2.5-15 µM N,N,N′,N′-tetrakis(2-pyridinylmethyl)-1,2-ethanediamine (TPEN), an intracellular zinc chelator. Zinc sufficiency was restored using 25 µM zinc sulfate (ZnSO₄·7H₂O). Cell viability was assessed by the MTT assay, cell cycle progression was evaluated using propidium iodide-based flow cytometry, and protein expression levels (Notch1, pAKT, p21, Bid, Bad, Bax, Caspase-3, MTF1, Znt1, Zip10, and SOCS3) were analyzed by Western blotting. mRNA expression of SOCS3 was quantified to evaluate the effect of zinc depletion on inflammation via the Stat3 pathway.

Results

Treatment with TPEN led to a dose-dependent reduction in Notch1 and pAKT levels, resulting in decreased p21 expression and increased apoptosis through a caspase-mediated mechanism involving Bid, Bad, Bax, and Caspase-3. Zinc depletion lowered MTF1 levels, thereby affecting the expression of zinc transporters Znt1 and Zip10 and disrupting zinc homeostasis. Propidium iodide cell cycle analysis showed that severe zinc depletion (10 and 15 µM TPEN) caused G1 phase arrest and significantly increased cell death (p < 0.05). An inverse correlation (p < 0.05) was observed between higher TPEN concentrations and p21 levels. Mild zinc depletion (2.5 and 5 µM TPEN) had no significant effect on SOCS3 mRNA levels or cell viability compared to controls, suggesting an adaptive cellular response under mild deficiency.

Conclusions

Zinc status critically influences the viability of RD cells by modulating MTF1-mediated zinc transporter expression and the Notch1-PI3K/AKT-p21 signaling axis. Severe zinc depletion disrupts zinc homeostasis, downregulates p21, induces G1 cell cycle arrest, and triggers apoptosis through caspase activation, while mild depletion is well tolerated. These findings highlight the importance of zinc homeostasis in skeletal muscle cell survival and provide mechanistic insights into zinc-related muscle pathology.

## Introduction

Zinc is an essential type II micronutrient that supports vital cellular processes such as survival, growth, and differentiation. It acts as a cofactor for numerous enzymes and hormones, which is key to its biological activity [1]. In the human body, total zinc content ranges from 2 to 3 g, predominantly located in skeletal muscle (around 60%). Additional major stores include bone (roughly 30%), while smaller portions are distributed in the liver, skin (approximately 5%), and other tissues (2-3%) [2]. Zinc deficiency is a prevalent dietary problem that often accompanies many chronic diseases, making it a crucial element in understanding changes at the cellular and molecular level [3]. The multifactorial nature of zinc deficiency, with low dietary intake being predominant, is a fascinating area of study. Chronic diseases, such as gastrointestinal disorders, diarrhea, renal disease, sickle cell anemia, cirrhosis, and cystic fibrosis, are known to lead to suboptimal zinc status [4,5]. In addition, the zinc status of an individual is determined by the genetic composition, especially in zinc-specific malabsorption syndrome, acrodermatitis enteropathica, which is a rare autosomal recessive inherited disease [6].

All of these conditions ultimately lead to moderate-to-severe zinc deficiency, resulting in increased levels of glucocorticoids. This process causes thymic atrophy, accelerates apoptosis in thymocytes, and reduces lymphopoiesis [7,8]. Zinc deficiency compromises the immune response. In contrast, myeloid progenitor cells exhibit enhanced cycling, with a higher percentage of cells in the S or G2/M phases noted for both MZD (33%) and SZD (56%) mice compared to zinc-adequate mice [9]. Conversely, zinc deficiency in neuroblastoma IMR-32 cells arrests the cell cycle at the G0/G1 phase and induces apoptosis [10]. Interestingly, immune system cells can adapt to the stress of suboptimal zinc levels by changing the expression of genes related to cytokines, DNA repair enzymes, and signaling molecules [11]. The cytokines produced during zinc depletion significantly impact tissues via the JAK/STAT pathway [12]. Consequently, STATs are regulated by the inhibitory action of SOCS3 [13]. Therefore, zinc levels ultimately determine physiological status and cell viability.

Endogenous zinc levels are shown to be pivotal in inducing autophagy under conditions of oxidative stress. Autophagy, a necessary preceding event for lysosomal membrane permeabilization and cell death in oxidative injury [14], is affected by zinc levels. Zinc depletion or lowering of intracellular zinc with chelating agents such as N,N,N′,N′-tetrakis(2-pyridinylmethyl)-1,2-ethanediamine (TPEN) blocks vacuole formation and zinc accumulation in the vacuole, inhibiting lysosomal activation and lysosomal membrane permeabilization [15]. Furthermore, zinc depletion in fibroblasts and neuroblastoma cells leads to apoptosis [16,17]. In contrast, high levels of zinc, achieved through exogenous zinc supplementation, have a contrasting effect. They inhibit prostate cancer initiation and/or progression by inducing cell cycle arrest, programmed cell death, or necrosis [18-20]. This contrast underscores the complexity of zinc’s role in cancer progression.

The role of zinc in chemotherapy revealed that supplemental zinc sensitized prostate cancer cells to paclitaxel-induced apoptosis. Conversely, this effect of paclitaxel was reduced at lower zinc levels (~8 μM) [20]. It is well known that p21 determines and regulates the cell cycle progression. P21 mRNA was upregulated in human prostate carcinoma cell lines when treated with zinc [19]. Moreover, the promoter region of p21 consists of potential metal-responsive elements (MREs), further supporting zinc regulation of p21 expression levels [21]. In addition, Akt and p-Akt ratio regulate the localization of p21 and influence the assembly and activity of the cyclin D-CDK4 complex during cell cycle progression through G1 to S phase [22,23]. Consequently, p21-sustained activation leads to cellular senescence [24].

It is important to note that zinc levels regulate P21 expression via Notch1 signaling along with the PI3K/AKT pathway [25]. Zinc transporters Znt1 and Zip10 play a crucial role in maintaining zinc homeostasis to zinc status in muscle [26]. Notably, metal transcription factor regulates these transporters whose expression is zinc-dependent [27]. Notch1 and its associated protein RBP-JK regulate the p21 expression upon translocation into the nucleus [25]. All these proteins are regulated by the intracellular zinc levels. Despite their significant role in cell viability, there is a notable scarcity of literature on the effects of zinc depletion on muscle cells. This study aims to elucidate the role of zinc transporters and the metal-regulatory transcription factor 1 (MTF1)-Notch1-p21 axis in TPEN-induced cell death in human rhabdomyosarcoma (RD) cells, with a focus on cell viability, cell cycle progression, and SOCS3-mediated inflammatory response under zinc-deficient conditions. Specifically, we aim to understand its impact on cell cycle progression, cell viability, and SOCS3 levels in zinc depletion and repletion conditions, thereby contributing to the existing body of knowledge in this field.

## Materials and methods

Rhabdomyosarcoma cell culture and maintenance

Human RD cells were procured (ATCC, Manassas, USA) and cultured in DMEM medium containing 10% fetal bovine serum, 1% glutamax, and 1% antibiotic-antimycotic (Life Technologies, California, USA). RD cells were meticulously maintained in a logarithmic growth phase, ensuring their health and a concentration between 0.6 and 0.8 x 10^6^ cells/mL. Media was changed every two days, and splitting was done at a 1:2 ratio when cells attained 70% confluence.

Cell culture conditions, viability, and TPEN chelation

All reagents were passed through a 0.22 µM sterile filter just before use. Cells were cultured and maintained at 37°C within a meticulously controlled, humidified environment containing 5% carbon dioxide. This setup ensures optimal conditions for cellular growth and functionality. Cells were cultured in a logarithmic growth phase at a concentration between 10^5^-10^6^ cells/mL. Cell viability was assessed by the dye exclusion method. Briefly, cells were harvested, washed with phosphate-buffered saline (PBS), and then cells were diluted to 1:10 using a 0.4% trypan blue solution. The cells were incubated for one to two minutes at room temperature, and the viable cells were examined at 10× magnification. Zinc chelation of TPEN was confirmed with the zinquin ethyl ester dye. Cells treated with TPEN were incubated with the zinquin ethyl ester, which emits fluorescence upon complexing with zinc (EVOS FL-Life Technologies).

Zinc supplementation and depletion (TPEN) treatments

Zinc sufficiency conditions were created by exposing the RD cells with 25 µM of zinc as zinc sulfate complexed with bovine serum albumin (BSA) in serum-free medium for four hours [28]. A range of zinc deficiency was created by treating the RD cells with increasing concentrations of TPEN (2.5 to 15 µM) (Sigma, St. Louis, MO, USA), as an intracellular zinc chelator, in serum-free media for four hours. The vehicle controls were BSA for zinc sufficiency and DMSO for zinc deficiency (as TPEN solubilized in DMSO). Each treatment was conducted in three separate independent experiments.

Cell viability

Cell viability was evaluated using the MTT assay (3-(4,5-dimethylthiazol-2-yl)-2,5-diphenyltetrazolium bromide; Himedia), following the manufacturer’s instructions. Briefly, RD cells (approximately 10,000 per well) were exposed to varying concentrations (2.5-15 μM) of zinc or TPEN, a chelator of intracellular zinc, for four hours in a serum-free medium [29]. After treatment with either zinc sulfate or TPEN, 10 μL of a 5 mg/mL MTT solution was added to each well, and the plate was incubated in the dark at room temperature for four hours. The supernatant was then removed, and the formazan crystals formed were dissolved in 100 μL of DMSO. Absorbance was recorded at 570 nm using a Vmax microplate reader (Synergy HT, BioTek, Winooski, USA). Cell viability was calculated using the following formula: percentage viability = ((A-B)/(C-B)) × 100, where A is the absorbance of the treated sample, B is the absorbance of the medium only, and C is the absorbance of the untreated control cells.

Cell cycle analysis

To induce varying levels of zinc deficiency, RD cells were treated with different concentrations (2.5-15 μM) of TPEN, an intracellular zinc chelator. After treatment, the adherent RD cells were detached using trypsin, centrifuged to collect the cells, and resuspended in ice-cold PBS. The cells were then fixed in absolute methanol for a minimum of 30 minutes. After fixation, they were washed with PBS containing 0.5% Tween-20 (Sigma, St. Louis, MO, USA), followed by two additional washes in PBS supplemented with 2% fetal calf serum (FCS). The cells were then incubated at 37°C for 20 minutes in PBS with 2% FCS and RNase-A at a concentration of 40 mg/mL. After this incubation, the cells underwent another PBS/FCS wash and were finally suspended in PBS containing 20 mg/mL propidium iodide (PI).

PI was used to stain the nuclei, allowing for the determination of DNA content. The fluorescence intensity of PI correlates with the stages of the cell cycle during cell division. PI fluorescence, which reflects the relative DNA content per cell, was measured using a flow cytometer (BD FACS Aria-II, New Jersey, USA). The FlowJo 10.7 software (Ashland, Oregon, USA) was utilized to calculate cell cycle distributions by categorizing relative DNA content per cell into sub-G1, G1, S, and G2/M fractions. Cell cycle distribution was based on 2N and 4N DNA content, where cells with more than 2N DNA content indicated apoptotic cells.

Detection of Notch1, AKT, MTF1, p21cip/kip, Bcl2 family, Caspase3, Stat3, and Socs3 using Western blot analysis

Cell lysis was performed using RIPA buffer supplemented with a protease inhibitor cocktail. Protein levels in the resulting supernatant were quantified using the micro-BCA assay. Equal amounts of protein (30 μg) were loaded onto 10-12% sodium dodecyl sulfate-polyacrylamide gel electrophoresis gels for separation and subsequently transferred to nitrocellulose membranes (Bio-Rad, California, USA). The membranes were then blocked with 5% non-fat dry milk and incubated overnight at 4°C with primary antibodies (Appendices). After extensive washing, the membranes were incubated with a horseradish peroxidase-conjugated IgG secondary antibody for one hour. Protein bands were visualized using an enhanced chemiluminescence detection kit (Bio-Rad), and images were acquired with the G-box imaging system (Syngene, USA). GAPDH served as a loading control to ensure equal protein input across samples.

Quantitative real-time polymerase chain reaction

Total RNA was extracted from the cells using Trizol reagent (Life Technologies), following the manufacturer’s guidelines. For cDNA synthesis, 1 μg of RNA was reverse transcribed using the Verso cDNA synthesis kit (ThermoFisher Scientific, California, USA). Quantitative real-time polymerase chain reaction (qRT-PCR) was performed using 2× SYBR Green Master Mix (Takara Bio, Shiga, Japan) with gene-specific primers (Appendices) on a BIO-RAD CFX 96 thermal cycler (C1000 model, Hercules, CA, USA). Each reaction was performed in duplicate to ensure reproducibility. PCR products were run on ethidium bromide-stained agarose gels to confirm the presence of a single, specific amplification product. GAPDH was used as the housekeeping gene for normalization due to its consistent expression across different conditions and cell types.

To further ensure specificity, melting curve analysis was performed on all qPCR products to confirm the amplification of a single target. A negative control lacking reverse transcriptase (no-RT control) was included to rule out genomic DNA contamination. The relative expression levels of target genes were calculated using the 2^-∆∆CT method.

Statistical analysis

Results are expressed as the mean ± standard error, based on a minimum of three independent experiments (n = 3), each including three technical replicates. Statistical analyses were performed using GraphPad Prism. Differences between control and treated groups were assessed using one-way analysis of variance, followed by Dunnett’s post hoc test for multiple comparisons. A p-value <0.05 was regarded as statistically significant.

## Results

Zinc and TPEN effect on cell viability

**Figure 1:**
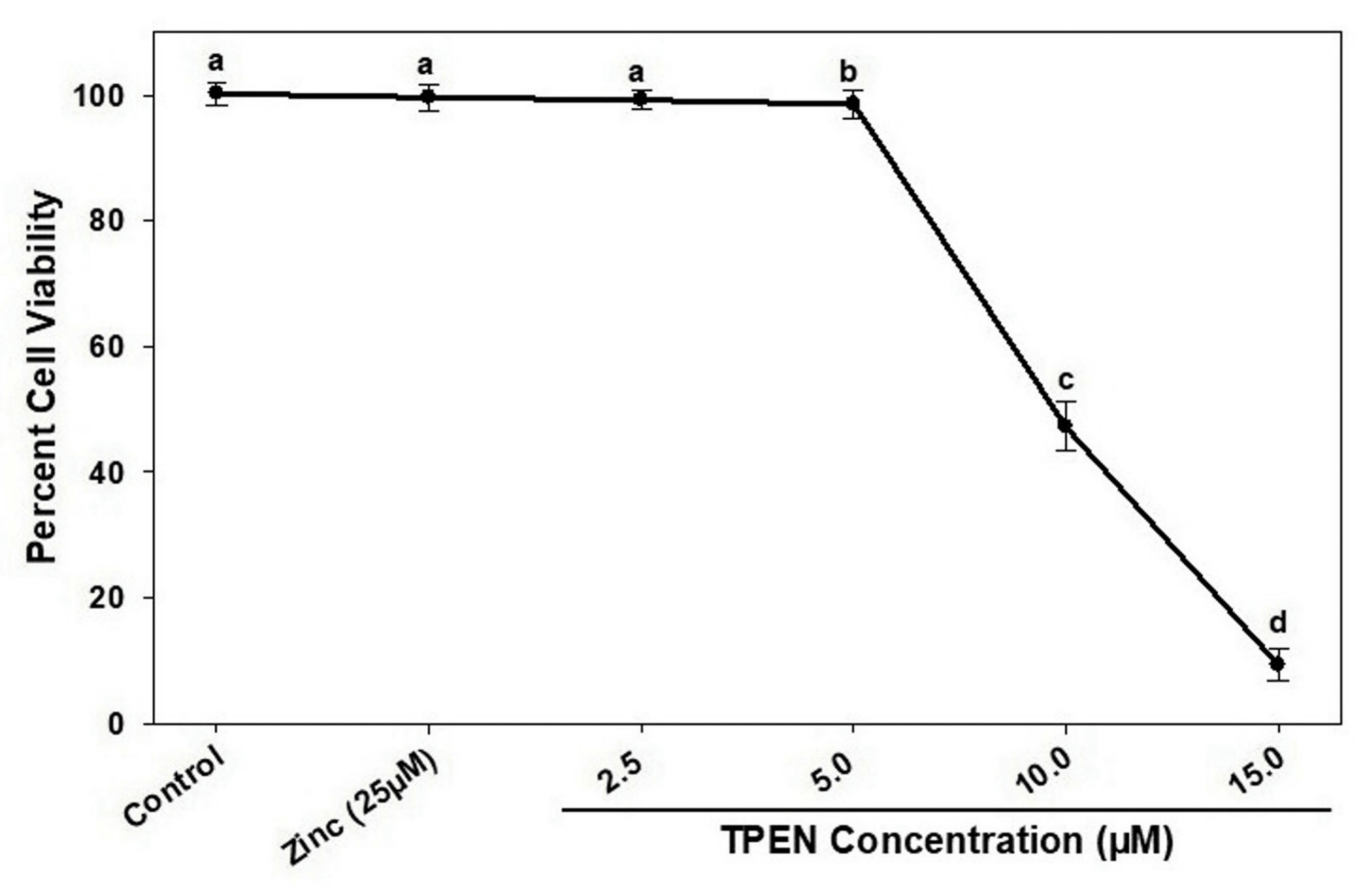
Cell viability: MTT assay. Cell viability was determined by the MTT assay. Rhabdomyosarcoma cells were incubated with the indicated concentration of either zinc or TPEN (T) in serum-free medium for four hours. Results are expressed as percentage viability, with 100% viability of control cells. Data sets are mean ± SE of n = 6 samples. Different superscripts are significantly different at a p-value <0.05.

The viability of RD cells was assessed following treatment with a concentration of 25 μM zinc and different levels of TPEN, specifically ranging from 2.5 μM to 15 μM. This evaluation was conducted over a four-hour period in a serum-free culture medium. The results of this investigation are illustrated in Figure 1. No significant cytotoxic effects on RD cell viability were observed following exposure to 25 μM zinc sulfate (Figure 1). Similarly, treatment of RD cells with 2.5 μM TPEN did not produce any noticeable changes in viability. In contrast, 5 μM TPEN demonstrated lower cytotoxicity, with 98.5% of the cells remaining viable compared to the control group. However, a significant increase in cytotoxicity (p < 0.05) was noted at higher concentrations of TPEN, with viability dropping to 47.19% at 10 μM and 9.2% at 15 μM, compared to the control (Figure 1).

Zinc deprivation on cell cycle progression in rhabdomyosarcoma cells

**Figure 2:**
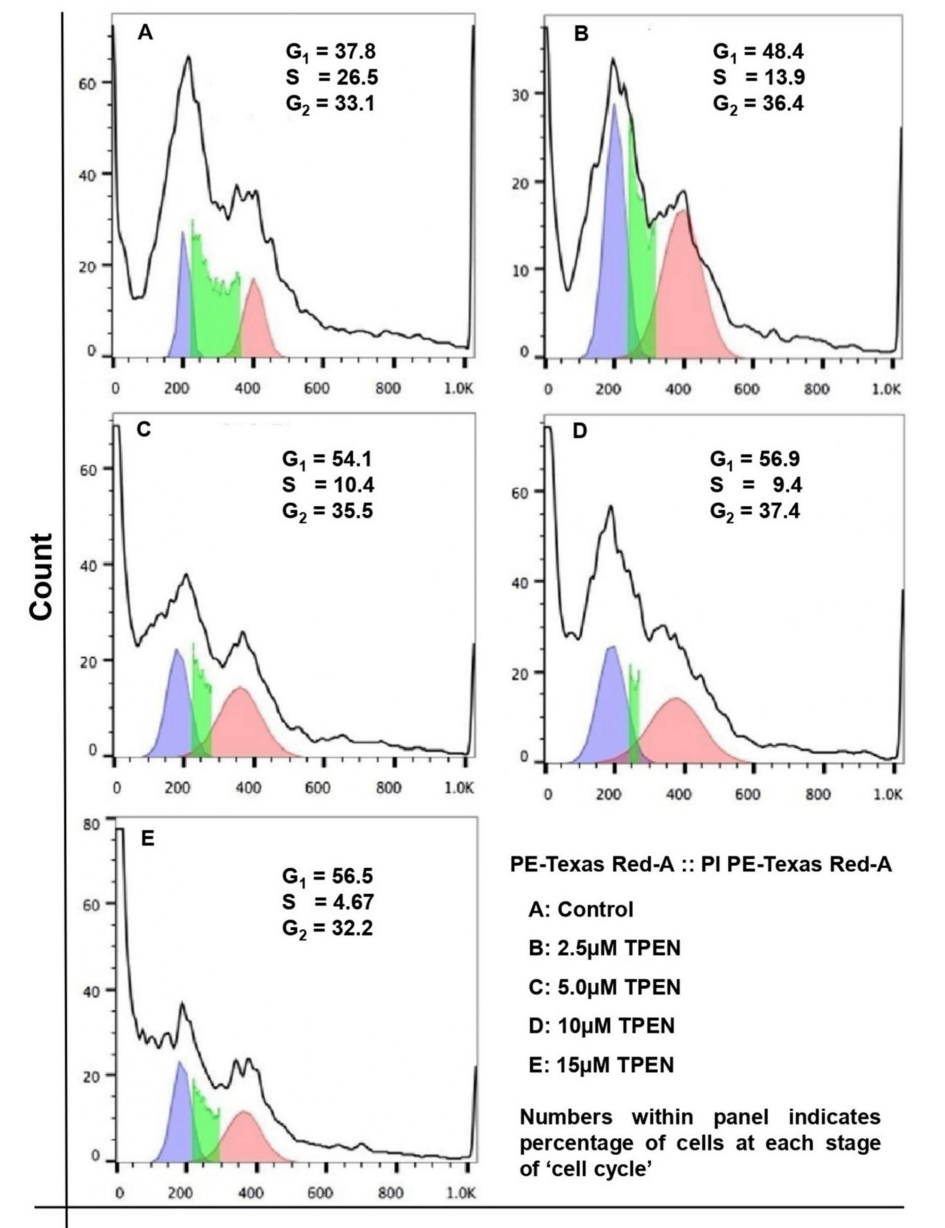
Cell cycle histogram of rhabdomyosarcoma (RD) cells. The cell cycle distribution pattern of zinc depletion, i.e., treatment of RD cells with TPEN (2.5 to 15 μM) (A) control (B) 2.5 μM TPEN (C) 5 μM TPEN (D) 10 μM TPEN and (E) 15 μM TPEN followed by subjecting for cell cycle analysis through flow cytometry with propidium Iodide. The numbers in each panel are percentage of cells at each stage of treatment.

The cell cycle stages of human RD cells, treated with TPEN (an intracellular zinc chelator) at a dose range of 2.5-15 μM for four hours, are presented in Figure 2. RD cells were arrested in the G1 phase compared to control, and the number of cells increased in the G1 phase with the extent of zinc depletion from 2.5 to 15μM TPEN, as shown in Table 1. Notably, a low dose of TPEN (2.5 μM) resulted in the arrest of 48% of cells in the G1 stage, while 5 µM TPEN led to the arrest of 58% of cells in the G1 stage compared to the control. Furthermore, higher concentrations of TPEN (10 and 15 µM) did not show a dose-dependent increase, except for 56% of cells at the G1 stage compared to controls (Table 1; Figure 2).

**Table 1:** Zinc depletion (TPEN treatment) effect on rhabdomyosarcoma cell cycle. Values indicate the percentage of cells in different stages of cell cycle treated with varying concentrations of TPEN (2.5-15 µM TPEN).

Treatment	Percentage of cells in different stages of the cell cycle		
	G_1_	S	G_2_/M
Control	37.8	26.5	12.6
2.5 µM TPEN	48.4	13.9	22.9
5 µM TPEN	54.1	10.4	26.8
10 µM TPEN	56.9	9.4	22.8
15 µM TPEN	56.5	4.7	18.8

Zinc deprivation on P21, Bcl2 family, and Caspase-3 expression in rhabdomyosarcoma cells

**Figure 3:**
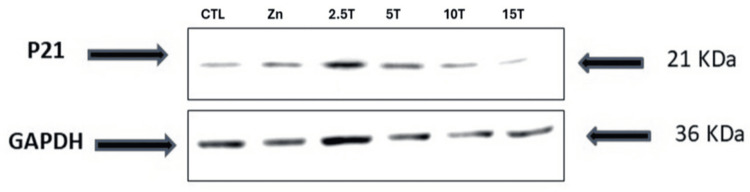
Effect of zinc status on p21. Rhabdomyosarcoma cells were treated with either zinc (25 µM ZnSO_4_·7H_2_O) or zinc chelator, i.e., TPEN (2.5-15 μM) for four hours in serum-free medium. Cells were lysed, protein concentration determined, and 30 μg protein was separated on 10% sodium dodecyl sulfate-polyacrylamide gel electrophoresis followed by transfer onto nitrocellulose membranes. The p21 protein levels were determined on membrane with anti-p21 antibody and horseradish peroxidase-conjugated IgG secondary antibody. The GAPDH was used as a reference control for equal loading of proteins. Zn is zinc and T is TPEN.

**Figure 4:**
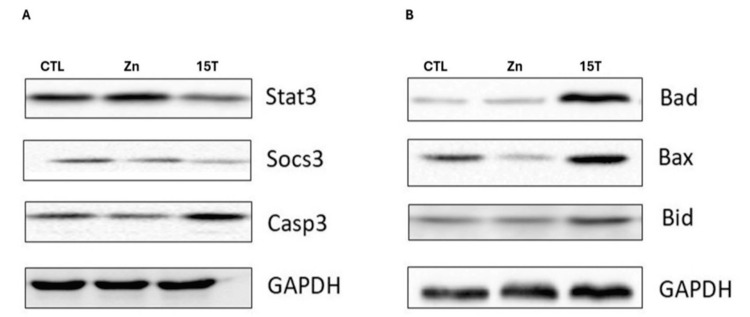
Effect of zinc status on Caspase-3, Bid, Bad, Bax, Stat3, and Socs3. RD cells were treated with either zinc (25 µM ZnSO_4_·7H_2_O) or zinc chelator, i.e., TPEN (2.5-15 μM) for four hours in serum-free medium. Cells were lysed, protein concentration determined, and 30 μg protein was separated on 10% sodium dodecyl sulfate-polyacrylamide gel electrophoresis followed by transfer onto nitrocellulose membranes. (A) Stat3, Socs3, and Caspase-3. (B) Bad, Bax, and Bid. The protein levels were determined on membrane with anti-antibody and horseradish peroxidase-conjugated IgG secondary antibody. The GAPDH was used as a reference control for equal loading of proteins.

**Figure 5:**
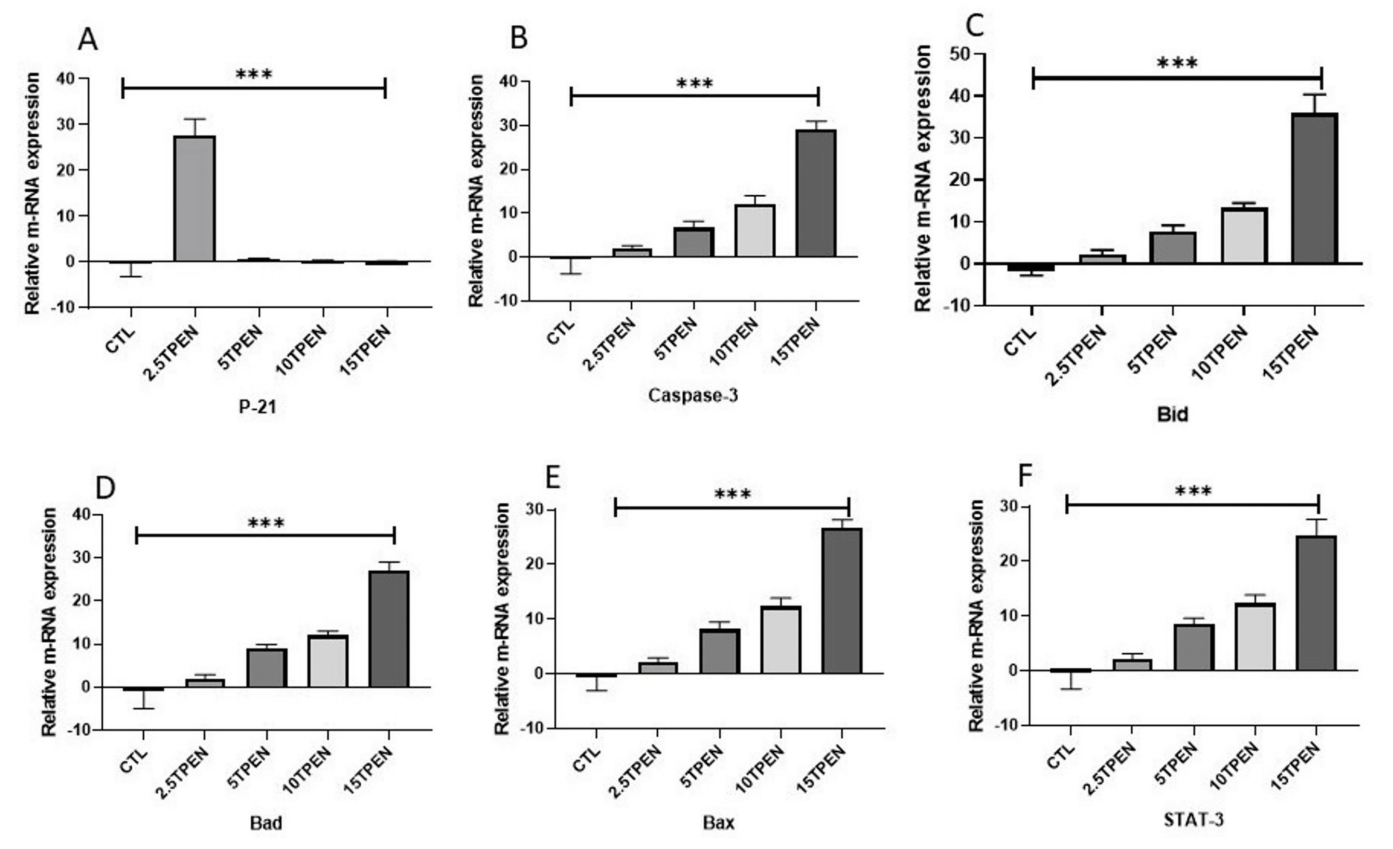
Effect of zinc status on p21 protein expression levels through caspase3 and Bcl2 family. The expression of P21, Bcl-2 family members, and Stat3 genes in rhabdomyosarcoma (RD) cells was evaluated after intracellular zinc was depleted using TPEN. Total RNA was extracted and reverse transcribed into cDNA. Quantitative polymerase chain reaction (qPCR) with SYBR green dye was employed to measure gene expression levels, and the resulting mRNA values were normalized to GAPDH. Panels A-F illustrate the relative mRNA expression of P21, Casapse-3, Bid, bad, Bax, Stat3, and ZnT in RD cells following treatment with TPEN at concentrations of 2.5 μM, 5 μM, 10 μM, and 15 μM (T is TPEN) for four hours. The data represent mean ± SE from three independent experiments, each performed in triplicate. Asterisks (*) denote statistically significant differences (p < 0.05). Statistical analysis was performed using analysis of variance, followed by Dunnett’s t-test for post hoc comparisons with the control group.

**Figure 6:**
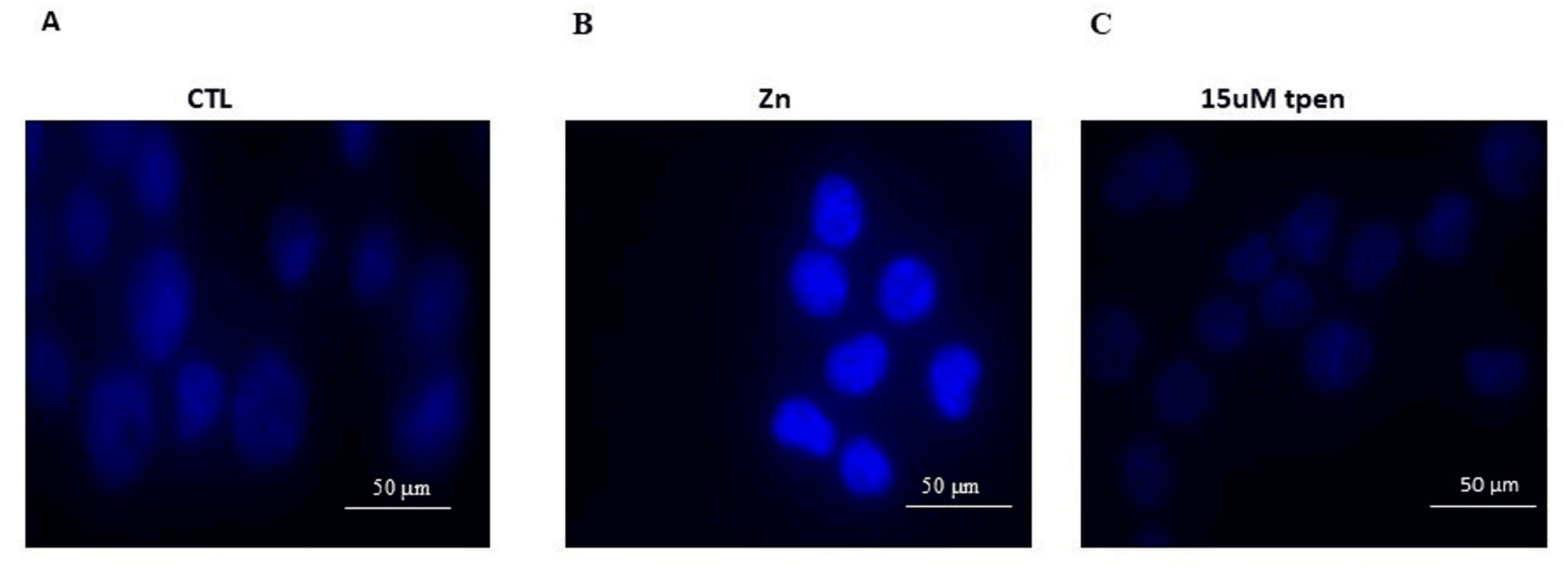
Effect of TPEN on intracellular zinc levels. To ensure that the TPEN created the zinc deficiency in rhabdomyosarcoma (RD) cells. Zinquin ethyl ester binds to the zinc and emits fluorescence upon binding zinc at 370 nm excitation and 420 nm emission. (A) Control. (B) Zinc-treated RD cells show bright fluorescence. (C) TPEN treated show weak fluorescence, confirming TPEN-induced zinc depletion. Zn is zinc and T is TPEN.

The p21 protein expression pattern of RD cells treated with different doses of TPEN, as shown in Figure 3, was meticulously studied. An inverse pattern of p21 protein expression was noted with increasing concentration of TPEN. A decrease in p21 level was observed with the increase in zinc depletion from 2.5 to 15 μM TPEN treatment. Further, a very low level of expression was observed in the 15 μM TPEN (Figure 3). When RD cells were treated with the higher dose of TPEN, it significantly elevated the Caspase-3 protein levels. Further, it elevated the BCl2 family proteins such as Bid, Bax, and Bad, both at RNA and protein levels (Figures 4, 5).To ensure the TPEN created the zinc deficiency in the RD cells, we used zinquin ethyl ester, which binds to the zinc and emits fluorescence upon binding with zinc (Figure 6).

Zinc deprivation on Stat3 and Socs3 expression in rhabdomyosarcoma cells

**Figure 7:**
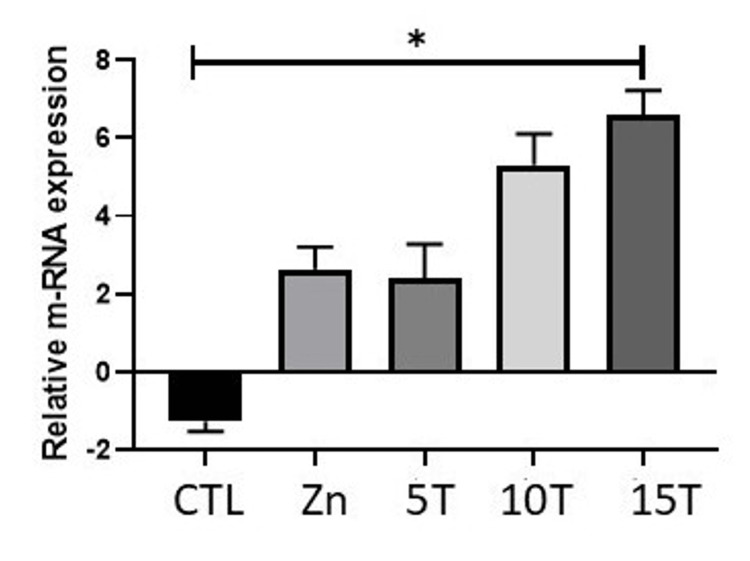
Effect of zinc status on SOCS3 expression levels through Stat3 mediated in rhabdomyosarcoma (RD) cells. Total RNA was extracted and used to synthesize complementary DNA (cDNA). RD cells were exposed to either Zn supplementation or 2.5 to 15 μM TPEN, a chelating agent that reduces intracellular zinc levels. Quantitative reverse transcription polymerase chain reaction was used to assess relative mRNA expression, with values normalized to GAPDH. Data are presented as mean ± SE. CTR represents the untreated control group, zinc corresponds to treatment with 25 μM zinc, and TPEN reflects zinc depletion via chelation. Statistical differences between treated and control groups were evaluated using analysis of variance, followed by Dunnett’s post hoc test for multiple comparisons. Zn is Zinc, and T is TPEN.

The SOCS3 mRNA levels of human RD cells treated with either 5 µM TPEN or 25 µM Zinc as zinc sulphate for four hours are presented in Figure 7. Although there was a slight decrease in SOCS3 mRNA levels in zinc sufficiency and vehicle control (BSA), they were comparable with the control (Figure 7). Similarly, RD cells treated with 5 µM TPEN and vehicle control (DMSO) showed lower levels of SOCS3 mRNA, but they were comparable with controls (Figure 7). However, when RD cells were treated with a high dose of TPEN, there was a significant reduction in the SOCS3 mRNA and protein levels, as shown in Figure 4. In contrast, Stat3, which regulates the SOCS3 expression, showed an opposite trend of expression with zinc treatment elevating its expression and TPEN reducing the expression (Figure 4), highlighting the complex regulatory mechanisms at play.

Zinc deprivation reduced Notch1, PAKT, MTF1, and zinc transporters

**Figure 8:**
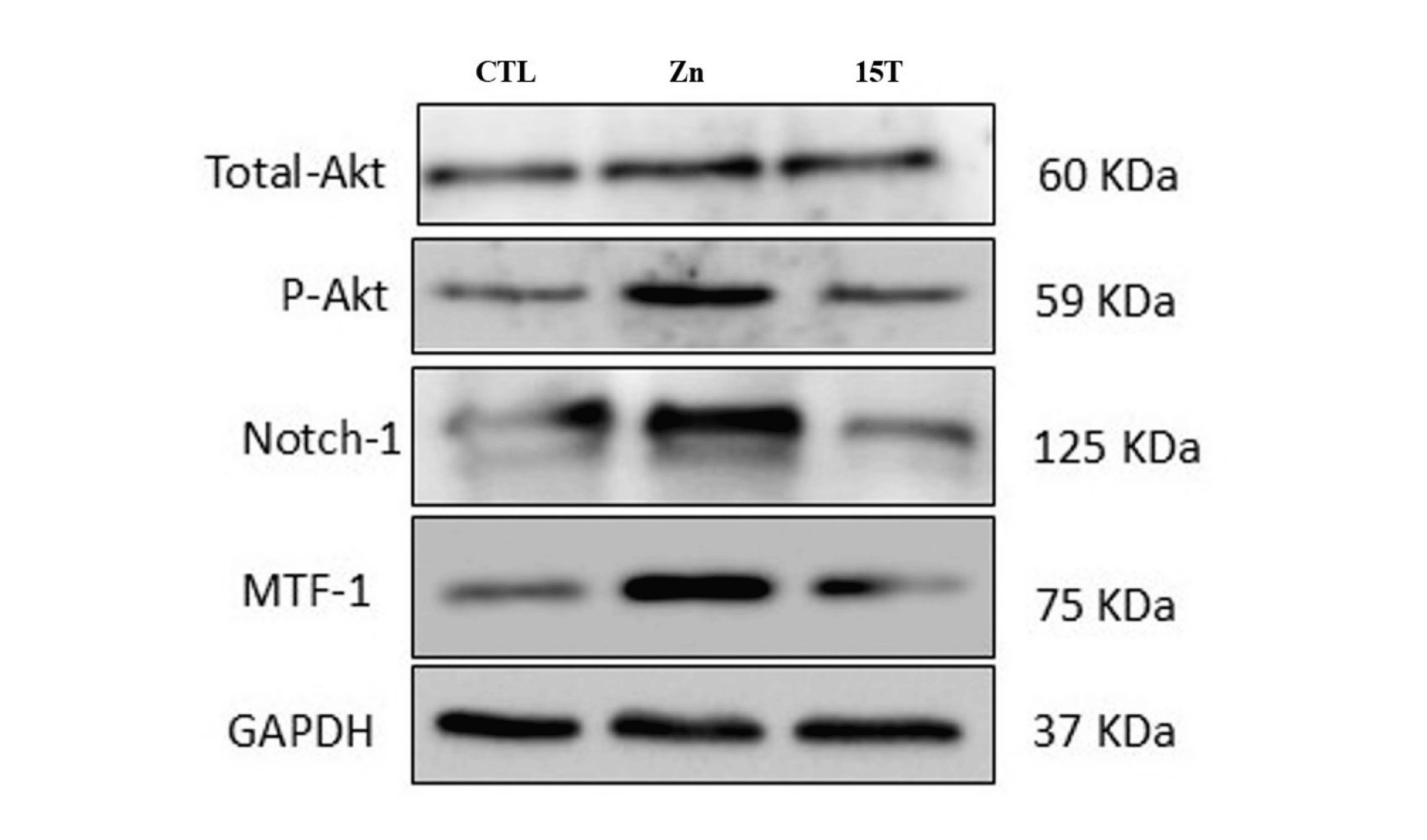
Effect of zinc status on Notch1, pAKT, and MTF1. Rhabdomyosarcoma cells were treated with either zinc (25 µM ZnSO_4_·7H_2_O) or zinc chelator, i.e., TPEN (15 μM) for four hours in serum-free medium. Cells were lysed, protein concentration was determined, and 30 μg protein was separated on 10% sodium dodecyl sulfate-polyacrylamide gel electrophoresis followed by transfer onto nitrocellulose membranes. The protein levels were determined on membrane with antibodies and horseradish peroxidase-conjugated IgG secondary antibody. The GAPDH was used as a reference control for equal loading of proteins. Zn is zinc and T is TPEN.

**Figure 9:**
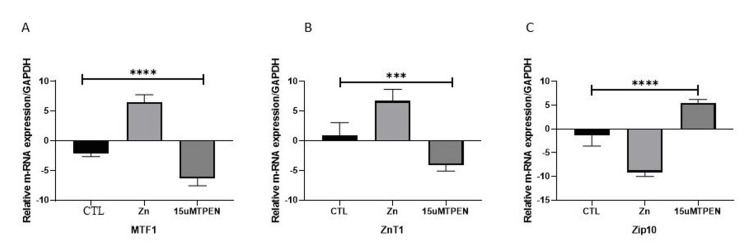
Effect of zinc status on MTF1-mediated Znt1 and Zip10 expression levels in rhabdomyosarcoma (RD) cells. RD cells were treated with either 25 μM zinc or 15 μM TPEN, a zinc chelating agent, followed by total RNA extraction and cDNA synthesis. Quantitative reverse transcription polymerase chain reaction was performed to evaluate relative mRNA expression, with normalization to GAPDH. Bar graphs display the mean ± SE of the measured mRNA levels. Treatment groups included: CTR (untreated control), zinc (25 μM zinc supplementation), and TPEN (zinc depletion via intracellular chelation). The analyzed transcripts included (A) MTF1, (B) Znt1, and (C) Zip10. Statistical significance between control and treated groups was determined using analysis of variance, followed by Dunnett’s post hoc test for multiple comparisons. Zn is zinc and T is TPEN.

The levels of PAKT and Notch1 proteins were significantly decreased in RD cells subjected to zinc deprivation induced by TPEN treatment (Figure 8). This reduction highlights the impact of zinc deficiency on these crucial regulators. Furthermore, the expression of zinc transporters revealed a contrasting pattern: Znt1, which is responsible for zinc uptake, showed a marked decrease, while the levels of Zip10, a transporter that facilitates zinc export, were significantly elevated (Figure 9). In addition, MTF1 regulated the expression of Znt1, and Zip 10 was also downregulated with zinc deprivation (Figure 8). This suggests a potential alteration in zinc homeostasis and transport mechanisms in response to the induced zinc deficiency.

## Discussion

Creating a controlled in vitro environment with a reduced zinc level model that accurately mimics in vivo conditions, particularly in terms of metabolic changes, hormones, cytokines, and other factors, is technically challenging. Additionally, the experimental process of inducing zinc deficiency may lead to compromised outcome parameters. In this study, we created an acute zinc depletion model using the intracellular chelator TPEN, and we examined its effects on cell viability and proliferation. Research suggests that disruptions in the signaling that govern cell cycle checkpoints or an inability to properly pause the cycle can trigger apoptosis, the process of programmed cell death [30]. In response to DNA damage, cells often arrest the cycle at either the G1/S or G2/M checkpoint. This interruption pauses progression, allowing time for DNA repair and preserving genomic stability by preventing the transmission of damaged DNA [31]. Interestingly, zinc depletion has similar effects on the cell cycle signalling molecules, thereby on the cell viability. Our findings showed that with an increase in the dosage of zinc depletion, RD cells were arrested in the G1 phase. Notably, at a high dose of TPEN (15 µM), approximately 56% of the cells were arrested in G1 compared to the control group (Figure 2). Therefore, our results confirm that zinc depletion influences the cell cycle process, even though muscle cells serve as the primary storage site for zinc.

It is concurrent that a severe form of zinc depletion (10 and 15 µM TPEN) affected the RD cell viability, along with arrest of a higher percentage (56%) of cells at the G1 phase. The MTT assay data revealed a clear pattern of cell death, with 53% of cells dying at 10 μM TPEN and a striking 90.8% at 15 μM TPEN (Figure 1). The contrasting results of the MTT and cell cycle assays suggest that mild zinc deficiency (low dose of TPEN) triggers a cell repair process, while severe zinc deficiency (higher concentrations of TPEN) impairs this process, leading to increased cell death.

In addition, the current study unveiled that the cell cycle arrest in RD cells at the G1 phase is through the hampering of p21 expression levels in relation to zinc depletion. It has been well known that p21 overexpression in cells induces G1, G2, or S phase arrest [32,33]. Whereas p21-deficient cells display a defect in DNA damage-induced G1 and G2 arrest [34,35]. Notably, the stabilization of p21 prevents apoptosis by inducing cell cycle arrest, thereby interacting with caspase-3 [36]. Mild DNA damage, as further evidenced from the MTT assay at lower concentrations of TPEN (2.5 and 5 µM), suggests that cells express p21 protein, which halts the cell cycle progression at the G1 stage, thereby repairing the damage and preventing cell death (Figure 3; Appendices). One potential explanation is that p21 could facilitate the transportation of its associated active cyclin-dependent kinase (CDK) complexes to sites of DNA repair by interacting with proliferating cell nuclear antigen. This interaction may play a crucial role in ensuring that the necessary proteins are delivered to the areas where repair is needed, thereby enhancing the efficiency of the repair process.

It is a well-established fact that at a higher degree of DNA damage (under unrepairable conditions), p21 protein undergoes cleavage, thus triggering apoptosis [37]. The current study findings are in line with this phenomenon, wherein RD cells treated with a high dose of TPEN (10 and 15 µM) created irreparable damage; hence, cells cleaved or degraded the p21 levels, as it prevents the cell cycle progression and makes way for cell repair (Figure 3). Once p21 is degraded, cells are allowed to enter the next stage of the cell cycle, as cells with DNA damage can no longer be repaired, leading to apoptosis or cell death. If this is the case, the cleavage of p21 by caspase-like activity could hinder the cell’s ability to effectively repair DNA damage. This disruption may lead to an accumulation of unresolved DNA lesions, which could ultimately activate the process of apoptosis, or programmed cell death. Our results on p21 degradation are in line with the literature and our MTT assay (Figure 1), which further supports our findings on the role of p21 in cell cycle arrest and apoptosis.

Our study focused on understanding the role of zinc in regulating Notch1 and P21 levels, particularly in the context of cancer biology. It is well established that zinc levels, regulated by the zinc transporters, influence the P21 levels by Notch1 signalling and PI3K/AKT pathways [25]. Our results with TPEN, a zinc chelator, showed that Notch1 levels were significantly reduced compared to controls. Also, the pAKT levels, whose levels impact the Notch1 proteosomal degradation, were reduced (Figure 8). Thus, TPEN-induced cell death in RD cells might be due to the reduced levels of pAKT, which are unable to prevent the proteosomal degradation of Notch1 signalling. Moreover, Notch1 specifically regulates the P21 expression upon complex with RJKB-p protein in the nucleus [25]. Thus, reduced levels of Notch1 and pAKT impact the P21 expression. Our results showed that pAKT levels reduced with TPEN might have a similar effect. Moreover, Notch1 levels are zinc-dependent, as the zinc transporters, which tightly regulate the zinc levels, are affected by TPEN. In addition, MTF1, whose activity controls the Znt1 and Zip10, was also reduced (Figures 8, 9). Thus, on the whole, zinc levels regulate the cellular physiology and viability via the MTF1-Notch1 axis in RD cells.

Our research has provided clear evidence that the degradation of P21 occurs through a mechanism activated by caspase-3. This well-established process is responsible for stimulating apoptosis, which involves the elevation of Bcl-2 family proteins such as Bid, Bad, and Bax [37]. Furthermore, the release of cytochrome C from the mitochondria triggers the caspase cascade, leading to the activation of caspase-3, which subsequently degrades P21. Our results have unequivocally confirmed this mechanism. Importantly, we have also demonstrated that zinc depletion operates through a similar apoptosis mechanism (Figure 4).

Zinc depletion has a significant impact on the elevation of several inflammatory molecules, including IL-1 and IL-6, and the activation of STAT3 [38]. Notably, zinc levels upregulate SOCS3 expression via phosphorylation at the tyrosine residue and the activation of STAT3 [39]. This underscores the crucial role of zinc levels in determining the functions of STAT3. Our results, which show a significant elevation of STAT3 in both zinc supplementation and zinc depletion, are in line with these studies (Figure 4). Our earlier findings have shown lower basal expression of SOCS3 levels in RD cells [40]. In the current study, mild Zinc deficiency created by 5 μM TPEN showed no significant effect on the SOCS3 mRNA levels in RD cells, whereas higher TPEN reduced the SOCS3 expression (Figure 7). Conversely, protein levels of SOCS3 were also reduced, which emphasizes that acute high zinc depletion has a total effect on the SOCS3 levels via STAT3 mediation (Figure 4). In contrast, zinc inhibits the STAT3 activation during the Th17 development [41].

SOCS3 has been shown to function as a proinflammatory mediator by suppressing IL-6-gp130 signalling, interfering with its ability to inhibit lipopolysaccharide (LPS) signalling [42]. These key findings provide a clear and comprehensive understanding of the complex interplay among zinc, STAT3, and SOCS3, and their implications for inflammatory responses. It is evident from animal experiments that mice lacking SOCS3 in macrophages and neutrophils are resistant to LPS-induced shock [42]. Moreover, accumulating data suggest that SOCS3 may suppress inflammatory responses [43]. Thus, the function of SOCS3 during inflammation seems to be dependent on the disease model used and the cell type studied. Our published results on RD support the tissue or cell specificity of zinc transporters according to the intracellular zinc levels and, in turn, affect the SOCS3 expression [40].

This study has identified several key limitations that could significantly impact future research in the field. These include the lack of data on p21 degradation into p14 and the caspase-cascade pathway, as well as the unclear mechanism of Notch1 activation and degradation in zinc repletion and zinc depletion conditions. The role of the RBK-Jp protein in forming the complex with the Notch1-IC under mild-to-severe zinc depletion is another area that requires further exploration. Additionally, investigating STAT3 protein levels upon using the inhibitor to show the SOCS3 mRNA levels in the severe form of zinc depletion could lead to important discoveries. Furthermore, knockout and overexpression studies of these proteins will enhance the mechanistic understanding of the micronutrient zinc regulation in muscle physiology.

## Conclusions

Zinc, a type II nutrient, is primarily stored in muscle. When RD (muscle) cells were treated with TPEN, an intracellular zinc chelator, the cells experienced an arrest in the G1 phase of the cell cycle, highlighting the crucial role of zinc in cell cycle progression. This zinc depletion not only interfered with cell cycle progression but also triggered cell death due to the maintenance of low p21 protein levels, which are essential for regulating the cell cycle. The interaction between the MTF1-Nocth1 signaling pathway and zinc transporters plays a crucial role in governing cell viability, underscoring the importance of zinc homeostasis in muscle cell health and function.

## Figures and Tables

**Figure 10:**
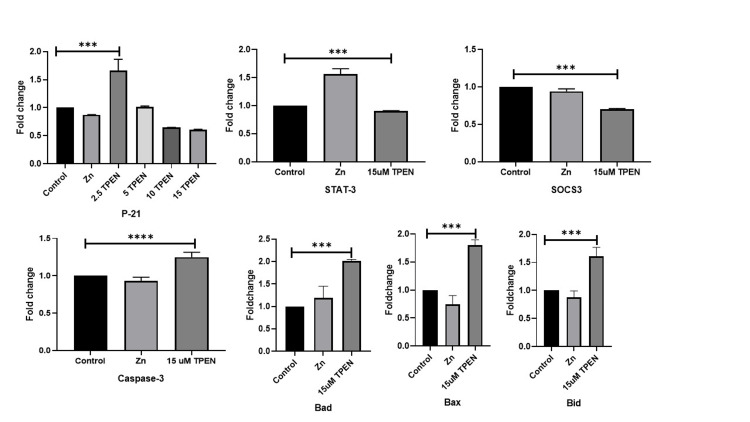
Image J analysis of the Western blot. Bar graphs represent the p21, Stat3, Socs3, Bid, Bad, and Bax proteins. Statistical significance between control and treated groups was determined using analysis of variance, followed by Dunnett’s post hoc test for multiple comparisons. Zn is zinc. Asterisk (*) represents the statistical significance.

## Appendices

**Table 2:** Primer sequences used for gene expression.

Sequence name	Primer
ZnT1F	TGTGAACTTGCCTGCAGAAC
ZnT-1R	TGGGGTCTTTTCTGCATCCT
ZIP-10F	CACAGTCACCAACATGCACA
ZIP-10R	TGCCTCCTAGAGCAACAA
GAPDH-F	GGATTTGGTCGTATTGGG
GAPDH-R	GGAAGATGGTGATGGGATT
PERK-F	GTCCCAAGGCTTTGGAATCTGTC
PERK-R	CCTACCAAGACAGGAGTTCTGG
CHOP-F	GGTATGAGGACCTGCAAGAGGT
CHOP-R	CTTGTGACCTCTGCTGGTTCTG
ATF6-F	CAGACAGTACCAACGCTTATGCC
ATF6-R	GCAGAACTCCAGGTGCTTGAAG
Stat3-F	GGGCATTTTTATGGCTTTCAAT
Stat3-R	GTTAACCCAGGCACACAGACTTC
P21-F	TGGAGACTC TCAGGGTCGAAA
P21-R	GGCGTTTGGAGTG GTAGAAATC
Bid-F	AGGAGAAGACCATGCTGGTG
Bid-R	CTCACGTAGGTGCGTAGGTT
Bad-F	TCACAAATCCTCCCCAAGTGG
Bad-R	GAATGCGCCCTAAATCACTGA
Bax-F	ACC CGG TGC CTC AGG ATG CGT
Bax-R	ACCCGGTGCCTCAGGATGCGT
Socs3-F	CCACTCTTCAGCATCTCTGT
Socs3-R	AGCTGGGTGACTTTCTCATA

**Table 3:** Antibodies used for Western blot.

Antibody name	Catalog and company
P21	#2947-Cell Signaling Technologies
Stat3	#12640-Cell Signaling Technologies
Bid	#2002-Cell Signaling Technologies
Bad	#9292 -Cell Signaling Technologies
Bax	#2772-Cell Signaling Technologies
Caspase-3	# 9662-Cell Signaling Technologies
Notch1	#3608-Cell Signaling Technologies
P-Akt	#9271- Cell Signaling Technologies
T-Akt	#4691-Cell Signaling Technologies
Socs3	#52113-Cell Signaling Technologies
MTF-1	#ab236401-Abcam
